# A hybrid design for dose‐finding oncology clinical trials

**DOI:** 10.1002/ijc.34203

**Published:** 2022-07-21

**Authors:** Jason J. Z. Liao, Feng Zhou, Heng Zhou, Lilli Petruzzelli, Kevin Hou, Ekaterine Asatiani

**Affiliations:** ^1^ Incyte Corporation Wilmington Delaware USA; ^2^ Merck & Co., Inc. North Wales Pennsylvania USA; ^3^ Genentech South San Francisco California USA; ^4^ Incyte Biosciences International Sàrl Morges Switzerland

**Keywords:** dose‐finding, dose‐limiting toxicity, hybrid design, MTD, overdosing toxicity, γ

## Abstract

Identifying the maximum tolerated dose (MTD) and recommending a Phase II dose for an investigational treatment is crucial in cancer drug development. A suboptimal dose often leads to a failed late‐stage trial, while an overly toxic dose causes harm to patients. There is a very rich literature on trial designs for dose‐finding oncology clinical trials. We propose a novel hybrid design that maximizes the merits and minimizes the limitations of the existing designs. Building on two existing dose‐finding designs: a model‐assisted design (the modified toxicity probability interval) and a dose‐toxicity model‐based design, a hybrid design of the modified toxicity probability interval design and a dose‐toxicity model such as the logistic regression model is proposed, incorporating optimal properties from these existing approaches. The performance of the hybrid design was tested in a real trial example and through simulation scenarios. The hybrid design controlled the overdosing toxicity well and led to a recommended dose closer to the true MTD due to its ability to calibrate for an intermediate dose. The robust performance of the proposed hybrid design is illustrated through the real trial dataset and simulations. The simulation results demonstrated that the proposed hybrid design can achieve excellent and robust operating characteristics compared to other existing designs and can be an effective model for determining the MTD and recommended Phase II dose in oncology dose‐finding trials. For practical feasibility, an R‐shiny tool was developed and is freely available to guide clinicians in every step of the dose finding process.

## Graphical abstract

What's new?

For novel cancer therapeutics, identifying the maximum tolerated dose (MTD) and the ideal dose for Phase II clinical trials are major goals of Phase I dose‐finding studies. Here, to better optimize dose selection, the authors present a hybrid approach to making dose estimates based on mode‐assisted design and a dose‐toxicity model. In a real trial example and simulation scenarios, the hybrid design limited the risk of overdosing toxicity and, owing to calibration for an intermediate dose, yielded a recommend dose more on par with true MTD. The results highlight the utility of the hybrid design for Phase I dose‐finding trials.

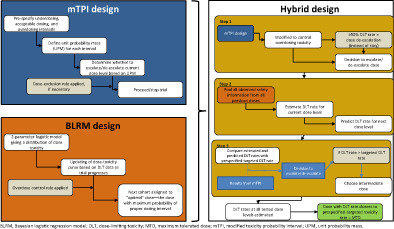

**Abbreviations**
BLRM: Bayesian logistic regression model
BOIN: Bayesian optimal interval
CRM: continual reassessment method
DL: dose level
DLT: dose‐limiting toxicity
EWOC: escalation with overdose control
MTD: maximum tolerated dose
mTPI: modified toxicity probability interval
PD: pharmacodynamic
PK: pharmacokinetic
UPM: unit probability mass

## 1 INTRODUCTION

The primary objective of a Phase I oncology dose‐finding clinical trial is to identify the maximum tolerated dose (MTD) of the investigational treatment and subsequently to recommend a dose for the dose‐expansion and Phase II trial. Typically, the MTD is defined as the highest dose with no more than a proportion of participants experiencing a dose‐limiting toxicity (DLT), and the fraction is typically denoted as the “target toxicity probability” (eg, 0.3). Finding this MTD correctly is crucial, as most responses occur at 80% to 120% of the MTD.^1^ Any dose below the true MTD can potentially lead to suboptimal efficacy and thus result in a negative Phase II or Phase III trial. Meanwhile, any dose above the true MTD can expose the participants to excessive toxicity.

Multiple trial design approaches have been developed for optimal dose‐finding of oncology therapeutics that can be classified into three categories. The first category is the algorithm‐based designs using a prespecified algorithm to sequentially decide the next dose for the treatment, such as the 3 + 3 design.^2^ The 3 + 3 design is simple and transparent, has fixed rules and is easy to use, leading to it being the most commonly used design for dose‐escalation trials in early years. However, there are many drawbacks to the 3 + 3 design: regardless of the true DLT rate, this design has the same fixed rules and fixed cohort size, with no reescalation allowed, and the 3 + 3 design is also poor at targeting the true MTD,^3^, ^4^, ^5^ resulting in a biased MTD estimate with high variability.^6^

Numerous novel approaches, in particular, Bayesian dose‐finding designs, have been developed to improve the accuracy in MTD identification, and their comparative performances have been studied extensively.^7^, ^8^ The second category is the model‐based designs, in which the design assumes a parametric model for the dose‐toxicity relationship and continuously updates the model parameters based on the accumulated data to guide dose escalation. The continual reassessment method (CRM) is the most well‐documented model‐based design,^9^ and multiple modifications have been proposed, including dose escalation with overdose control (EWOC)^10^ and the Bayesian logistic regression model (BLRM),^11^ among others. The model‐based designs have excellent operating characteristics, with full inference along with uncertainty assessments for true DLT rates. These designs allow flexible cohort sizes and a feasible selection of intermediate doses. However, executing these designs can be time‐consuming. Other limitations include the nontransparent rules for decision‐making, which sound like a “black box” to a nonstatistician, and robustness issues from model misspecification.

The third category is the model‐assisted designs, which are relatively new designs with more transparent dose transition rules and similar operating characteristics compared to the model‐based designs.^7^, ^8^ Examples of model‐assisted designs are the modified toxicity probability interval (mTPI) design^12^ and its variation, mTPI‐2,^13^ the Bayesian optimal interval (BOIN) design^14^, ^15^ and the keyboard design.^16^ These designs share the same rule transparency as the 3 + 3 design but improve the performance and provide certain rules regarding the cohort size. However, these designs could have unacceptable overdosing levels. The BOIN design uses an optimal interval, and deviation from this optimal interval can lead to a high variation in decisions. Another drawback shared with the 3 + 3 design is that the information from each dose level in these designs is treated independently; that is, the safety information from previous doses is ignored when making dose transition decisions, and available firsthand knowledge about the experimental treatment is wasted, reducing efficiency.

The mTPI design is a widely used model‐assisted design and has a robust performance regardless of the choice of the acceptable toxicity interval. However, the suboptimal overdose control of the mTPI design has been noticed by many practitioners^16^; for example, if the target DLT probability is 30%, the mTPI design fails to de‐escalate the dose when three DLTs are observed out of six participants, which is a 50% DLT rate. Similar case occurs for two DLTs out of four participants. The keyboard design intended to overcome this drawback with some success. However, the controlling overdosing toxicity using the keyboard design remains not very efficient. For example, using a beta(0.005, 0.005) prior and the target key or acceptable interval [0.27, 0.33] for the target DLT rate 30%, the keyboard design also fails to de‐escalate the dose when three DLTs are observed out of six participants or two DLTs are observed out of four participants. Thus, to address the overdosing risk more efficiently, we will consider some modifications first in the algorithm of the mTPI design to guarantee a dose de‐escalation when the observed toxicity rate is high. Also, we can impose stricter overdose control rules to eliminate the overly toxic doses and ensure that participants are not treated at such dose levels if overly high toxicity rates are observed. It is common practice to impose overdose control rules in the model‐based designs such as EWOC and the BLRM. Note that mTPI instead of keyboard design is chosen to be modified is due to only three intervals in mTPI. Keyboard design further divides the overdosing interval of mTPI into small equal length overdosing keys, which differentiates each overdosing key. However, this is not reasonable to clinicians since no matter whether it is overdosing Key 1 or overdosing Key 2, it should be considered overdosing and thus all these overdosing keys should be combined, as labeled as overdosing interval in mTPI.

Another limitation of the model‐assisted designs is that they can only be implemented in predefined dose levels.^17^ However, sometimes it may be necessary to test an intermediate dose level if the lower dose level is too low but the next higher dose level is expected to be too toxic. This can be easily accomplished in model‐based designs because the toxicity probability can be estimated at any given dose level with the dose‐toxicity curve.

In summary, the three types of existing designs have different merits and limitations. In this article, we propose a novel hybrid design to incorporate the desirable features of both model‐based and model‐assisted designs. The hybrid design was developed based on the infrastructure of the mTPI design, to keep the simplicity of dose transition rules at each individual dose level. An overdose control rule has been added to avoid treating too many participants at toxic dose levels. Meanwhile, a dose‐toxicity model, such as a logistic regression model, is used to pool all the available information from previous doses and account for a dose‐toxicity relationship, as used in the BLRM design, to more accurately estimate the toxicity at the current dose level and to allow the flexibility of adding intermediate doses during trial conduct. The operating characteristics of the hybrid design have been demonstrated through simulations and a real trial dataset.

## 2 METHODS

The proposed hybrid design incorporates the features of the mTPI and BLRM designs, which are briefly discussed below. Suppose there are J provisional dose levels of an investigational treatment, denoted by $d_{1}<\cdots <d_{J}$, with toxicity probabilities $p_{1}<\cdots <p_{J}$. Let $n_{j}$ and $y_{j}$ be the number of participants and number of observed DLTs at dose level $d_{j}$, respectively. We use ϕ to denote the target toxicity probability.

### 2.1 mTPI design

The mTPI design uses a beta‐binomial model at each dose level as follows:

$$y_{j}|n_{j},p_{j}\sim \text{Binom}\left(n_{j},p_{j}\right),$$

$$p_{j}\sim \text{Beta}\left(a,b\right).$$

Thus, the posterior distribution of toxicity probability is:

$$p_{j}|n_{j},y_{j}\sim \text{Beta}\left(y_{j}+a,n_{j}-y_{j}+b\right).$$

Given a target toxicity probability ϕ, the mTPI design prespecifies three intervals with parameters $\delta_{1}=\phi -\varepsilon_{1},\;\delta_{2}=\phi +\varepsilon_{2}$, that is, the underdosing interval $\left(0,\delta_{1}\right)$, the acceptable dosing interval $\left[\delta_{1},\delta_{2}\right]$ and the overdosing interval $\left(\delta_{2},1\right)$, where $0<\delta_{1}<\phi <\delta_{2}<1$. Then, the mTPI design defines a quantity named unit probability mass (UPM) given the posterior distribution of $p_{j}$ for each of the three intervals as follows:

$$\mathrm{UPM}1=\mathrm{Pr}\;\left(p_{j}<\delta_{1}|n_{j},y_{j}\right)/\delta_{1},$$

$$\mathrm{UPM}2=\mathrm{Pr}\;\left(\delta_{1}\leq p_{j}\leq \delta_{2}|n_{j},y_{j}\right)/\left(\delta_{2}-\delta_{1}\right),$$

$$\mathrm{UPM}3=\mathrm{Pr}\;\left(p_{j}>\delta_{2}|n_{j},y_{j}\right)/\left(1-\delta_{2}\right).$$

That is, the UPM is the posterior probability that $p_{j}$ lies in the corresponding interval divided by the length of that interval. The mTPI design determines dose escalation/de‐escalation only based on the observed data at the current dose level j as follows:

If $\mathrm{UPM}1=\max\left\{\mathrm{UPM}1,\mathrm{UPM}2,\mathrm{UPM}3\right\}$, escalate dose to level j+1;
If $\mathrm{UPM}2=\max\left\{\mathrm{UPM}1,\mathrm{UPM}2,\mathrm{UPM}3\right\}$, stay at the current dose level j;
If $\mathrm{UPM}3=\max\left\{\mathrm{UPM}1,\mathrm{UPM}2,\mathrm{UPM}3\right\}$, de‐escalate dose to level j−1.

Because the three UPMs can be calculated for all the possible outcomes of $n_{j}$ and $y_{j}$, dose escalation and de‐escalation rules can be determined before the onset of the trial. To avoid treating excessive participants at extremely toxic dose levels, the mTPI design implements a dose‐exclusion rule: if $\mathrm{Pr}\;\left(p_{j}>\phi |n_{j},y_{j}\right)>0.95$, dose level j and higher doses are excluded in the trial. If the lowest dose is excluded, the trial is stopped for safety.

### 2.2 BLRM design

The BLRM design utilizes a 2‐parameter logistic model:

$$\text{logit}\left(p_{j}\right)=\log\left(\alpha \right)+\beta \times \log\left(\frac{d_{j}}{d^{*}}\right),\;\alpha ,\;\beta >0,\;j=1,\;\ldots ,\;J,$$

where α,β are the unknown parameters and $d^{*}$ the prespecified reference dose. Usually, a vague bivariate normal distribution is assigned for the prior of $\log\left(\alpha \right),\log\left(\beta \right)$. During the trial conduct, BLRM updates the estimate of the dose‐toxicity curve based on the accumulated DLT data across all dose levels.

Similar to the mTPI design, the BLRM defines the proper dosing interval $[\delta_{1},\delta_{2}$] as the range of toxicity probabilities regarded as acceptable. The BLRM imposes an overdose control rule as follows: if the observed data suggest that there is more than 25% posterior probability that the DLT rate of a dose is greater than $\delta_{2}$, that is, $\mathrm{Pr}\;\left(p_{j}>\delta_{2}|n_{j},y_{j}\right)\geq 0.25$, that dose is an overdose and cannot be used to treat participants. Then, among the dose levels satisfying the safety criterion, the BLRM assigns the next cohort of participants to the “optimal” dose, which is defined as the dose with the maximum posterior probability of the proper dosing interval.

### 2.3 The hybrid design

The hybrid design is a hybrid of the mTPI design and a dose‐toxicity model in three steps.

#### 2.3.1 Step 1

The mTPI design is modified to control the overdosing toxicity using the posterior probability of the DLT rate in the overdosing interval $\left(\delta_{2},1\right)$ being less than a value *γ* (eg, less than 0.75). With this rule, if three DLTs are observed out of six participants, which is a 50% DLT rate, the modified mTPI design will guarantee a dose de‐escalation instead of staying at the current dose level when the observed toxicity rate is high. Table 1 shows the decision rules based on the modified mTPI design with 30% target toxicity rate, where the overdosing toxicity issue is removed.^12^, ^18^ Thus, the modified mTPI is very efficient in controlling the overdosing toxicity.

**TABLE 1:** Dose‐escalation rules per modified mTPI design with 30% target toxicity rate [Colour table can be viewed at wileyonlinelibrary.com]

No. of participants with ≥1 DLT	Number of participants evaluable for DLT						
	3	4	5	6	7	8	9
0	E	E	E	E	E	E	E
1	S	S	S	E	E	E	E
2	D	D	S	S	S	S	S
3	DU	DU	D	D	S	S	S
4		DU	DU	DU	D	D	D
5			DU	DU	DU	DU	DU
6				DU	DU	DU	DU
7					DU	DU	DU
8						DU	DU
9							DU

*Note*: Target toxicity rate *ϕ*: 30%. Flat noninformative prior beta(1,1) is used as a prior and *ε*_1_ = *ε*_2_ = 0.05.^12^, ^18^ Posterior toxicity probability cut : 0.75.

Abbreviations: D, de‐escalate to the next lower dose; DLT, dose‐limiting toxicity; DU, the current dose is unacceptably toxic; E, escalate to the next higher dose; S, stay at the current dose.

#### 2.3.2 Step 2

Since the mTPI design approach treats each dose level independently, the information from all previous doses is disregarded. In contrast, the second step of the hybrid design is to use a dose‐toxicity model by pooling all observed safety information from all previous doses to estimate the DLT rate for the current dose level and predict the DLT rate for the next dose level in the provisional dose list. For example, a frequentist logistic dose‐toxicity model could be used. The estimated DLT rate at the current dose level is used together with the decision rules from the earlier modified mTPI design (as shown in Table 1) to make a decision about dose escalation. Note that if the dose‐toxicity model is not feasible (eg, no DLT is observed in all tested doses), then no action is needed at this step.

#### 2.3.3 Step 3

If the decision following the modified mTPI design in Step 1 was to escalate to the next higher dose in the provisional dose list, then the predicted DLT rate using the dose‐toxicity model from Step 2 is used to judge if the next dose level is feasible or not by comparing the predicted DLT rate at the next dose level with the prespecified‐targeted DLT rate (Table 1). If the predicted DLT rate is over the targeted DLT rate, the next dose level in the provisional dose list cannot be used. Instead, an intermediate dose from the earlier dose‐toxicity model will be calibrated so that the DLT rate is closer to the targeted DLT rate. Similarly, if the decision was to de‐escalate to the next lower dose in the provisional dose list, an intermediate dose from the earlier dose‐toxicity model can be calibrated so that the DLT rate is closer to the targeted DLT rate if the toxicity at the next lower dose level is too low. Note that choosing an intermediate dose level should be clinically and operationally feasible (eg, based on tablet strength or expected pharmacokinetics [PK]). If the decision was to stay at the current dose, then the estimated DLT rate at the current dose using the dose‐toxicity model from Step 2 is used to make a decision. If the estimated DLT at the current dose is over the prespecified‐targeted DLT rate, then the decision is to dose de‐escalate, otherwise, it is to stay. Note that the number of additional participants required to avoid overtoxicity can be guided using the rule from the modified mTPI design in Step 1, which is another advantage of the hybrid design over the BLRM approach.

At the end of the dose‐escalation procedure, the DLT rates at all tested dose levels are estimated based on the dose‐toxicity model or the pool‐adjacent‐violators algorithm if the parametric dose‐toxicity model is not feasible. The dose with an estimated DLT rate closest to the prespecified‐targeted toxicity rate will be treated as a preliminary MTD. However, the totality of the available data, such as the emerging safety, PK, pharmacodynamic (PD) and other biomarker information, will be considered before deciding on the dose(s) to be carried forward to the next phase (eg, the cohort expansion phase or Phase II trial).

## 3 A TRIAL EXAMPLE

We considered a dose‐escalation trial to determine the MTD and/or recommended dose for expansion of an antibody *XYZ* against a target expressed on immune cells, administered every 14 days in participants with selected tumor types. During dose escalation, cohorts of participants were treated with *XYZ* until the MTD was reached or a lower recommended dose(s) was established. The dose escalation was guided by an adaptive BLRM following the EWOC principle. During the dose escalation, additional cohorts of up to six participants could be enrolled at any planned or intermediate dose level below the next dose level or the MTD to better characterize safety, PK and/or PD activity. The MTD was defined as the highest dose not expected to cause DLT in ≥33% of the treatment participants in the first 28 days of *XYZ* treatment during the escalation part of the trial. The provisional dose levels and the corresponding DLT numbers from the trial are listed in Table 2, where the third column shows the estimated DLT rate and predicted DLT rate using a logistic regression model. At the dose level (DL) 6, the BLRM algorithm led to an intermediate dose level at a dose level between DL5 and DL6.

**TABLE 2:** Dose levels and observed DLT numbers

Dose, mg	No. of participants with DLT/total no. of participants	Estimated P(DLT at current dose) or predicted P(DLT at next dose)
DL10		
DL9		
DL8		
DL7		
DL6	2/6	*P*(DLT at dose DL5.5) = .32, where DL5.5 is an intermediate dose between DL5 and DL6 *P*(DLT at dose DL6) = .355 *P*(DLT at dose DL7) = .854
DL5	1/5	
DL4	0/3	
DL3	0/1	
DL2	0/1	
DL1	0/1	

Abbreviations: DL, dose level; DLT, dose‐limiting toxicity; *P*, probability.

By way of comparison, the results from applying the hybrid design and other methods to determine the MTD are listed in Table 3. There were two DLTs out of six participants at DL6, thus, the 3 + 3 design gave an MTD at DL5. The dose‐toxicity model and the raw point estimate indicated that the DLT rate at DL6 was over the targeted 33%; however, both the mTPI design and the BOIN design led to a “stay at the current dose level” conclusion and continued to enroll additional participants at DL6. For the hybrid design, additional toxicity evaluation was added to the mTPI design rules. As shown in the third column in Table 2, the estimated DLT rate at the current DL6 and the predicted DLT level at next dose level (DL7) were over the 33% target toxicity; thus, the hybrid design led to a de‐escalation. However, the DLT rate at DL5 was too low; therefore, an intermediate dose level was recommended and the DLT rate at DL5.5 was estimated (Table 2), which was closer but still below the target toxicity of 33%. Screenshots of the hybrid design results using a developed R‐shiny tool and R‐code can be viewed in the Supplementary 1 and 2, respectively.

**TABLE 3:** MTDs from different trial designs

Trial design	MTD
3 + 3	DL5
mTPI	DL6 (stay at DL6 from the design chart)
BOIN	DL6 (stay at DL6 from the design chart)
BLRM	An intermediate dose level: >DL5 but <DL6
Hybrid	An intermediate dose level: >DL5 but <DL6

Abbreviations: BLRM, Bayesian logistic regression model; BOIN, Bayesian optimal interval; DL, dose level; MTD, maximum tolerated dose; mTPI, modified toxicity probability interval.

Table 3 indicates that the 3 + 3 design selected an undertoxic MTD, both the mTPI design and the BOIN design selected an overtoxic MTD, but the BLRM and the hybrid design selected a reasonably toxic MTD level between DL5 and DL6. Although the hybrid design reached a similar conclusion to the BLRM, the hybrid design implemented the logistic regression model in a frequentist setting, which did not require the Bayesian setting using a priori and the Markov Chain Monte Carlo simulations. Due to the unacceptable toxicity at current DL6, an intermediate dose level, DL5.5, below but close to DL6, was likely feasible.

## 4 NUMERICAL TRIAL

We conducted a simulation trial to compare the operating characteristics of the proposed hybrid design with the mTPI, BOIN, Keyboard design, CRM, BLRM and 3 + 3 designs at the target toxicity level of 0.20, 0.25 and 0.30. A total of 15 true toxicity scenarios are displayed in Table 4A. In each scenario, there were five dose levels, with the true DLT in bold. It was assumed that the toxicity level monotonically increased with dose level. The true DLT was placed at different dose levels. A cohort size of three was used for all methods so that the methods were comparable to the 3 + 3 design. The maximum number of participants that could be dosed was 30. A total of 10 000 trials were simulated for each scenario.

**TABLE 4:** (A) True toxicity scenarios; (B) true toxicity scenarios for intermediate dose as MTD

	
(A)	True DLT rate for each dose level				
Scenario	Dose 1	Dose 2	Dose 3	Dose 4	Dose 5
1	**0.20**	0.25	0.30	0.40	0.45
2	0.15	**0.20**	0.25	0.30	0.35
3	0.10	0.15	**0.20**	0.25	0.30
4	0.05	0.10	0.15	**0.20**	0.25
5	0.04	0.08	0.11	0.16	**0.20**
6	**0.25**	0.30	0.40	0.45	0.50
7	0.20	**0.25**	0.30	0.35	0.40
8	0.15	0.20	**0.25**	0.30	0.35
9	0.10	0.15	0.20	**0.25**	0.30
10	0.08	0.11	0.16	0.20	**0.25**
11	**0.30**	0.40	0.50	0.60	0.70
12	0.20	**0.30**	0.40	0.50	0.60
13	0.10	0.20	**0.30**	0.40	0.50
14	0.10	0.15	0.20	**0.30**	0.40
15	0.05	0.10	0.15	0.20	**0.30**

(B)						
Intermediate dose		True DLT rate for each dose level				
Scenario	Target DLT	Dose 1	Dose 2	Dose 3	Dose 4	Dose 5
1	0.125	0.10	0.15	0.25	0.35	0.45
2	0.20	0.10	0.15	0.25	0.35	0.45
3	0.25	0.20	0.30	0.40	0.50	0.60
4	0.30	0.10	0.15	0.25	0.35	0.45

*Note*: Bold text denotes target DLT.

Abbreviation: DLT, dose‐limiting toxicity.

In the 3 + 3 design, the dose‐escalation procedure usually stops before the sample size reaches 30, given the nature of the design. To make the average number of participants treated at the MTD comparable, the remaining participants were treated at the selected MTD for a 3 + 3 design. For the mTPI and hybrid designs, the proper dosing interval was set to $[\delta_{1},\delta_{2}$] = [*ϕ* − 0.05, *ϕ* + 0.05], where *ϕ* was the target DLT. For the Keyboard design, the target key was set to [*ϕ* − 0.05, *ϕ* + 0.05]. For the BOIN, an optimal interval was used and for BLRM design, a default dosing interval was used. As the BLRM and hybrid design require a dose‐toxicity relationship, dose levels of 3, 6, 12, 18, and 24 mg were assumed, corresponding to the toxicity levels for all scenarios shown in Table 4A. In the BLRM, 24 mg was used as the reference dose. A noninformative prior of (log *α*, log *β*)

logαlogβ∼N−0.8470.381,2.0152001.0272

was applied according to Neuenschwander et al.^11^ The CRM utilized a 1‐parameter power model with a normal prior of $\alpha \sim N\left(0,2\right)$.

The following metrics were used to demonstrate the operating characteristics of the six designs:

- Probability of correct selection: the number of trials with target dose selected as the MTD/10 000.
- Average participants treated at MTD: the average number of participants assigned to the MTD across 10 000 trials.
- Probability of overdosing: the number of trials with selected dose above the true MTD/10 000.
- Probability of underdosing: (the number of trials with selected dose under the true MTD + the number of trials terminated early)/10 000.

### 4.1 RESULTS

**FIGURE 1:**
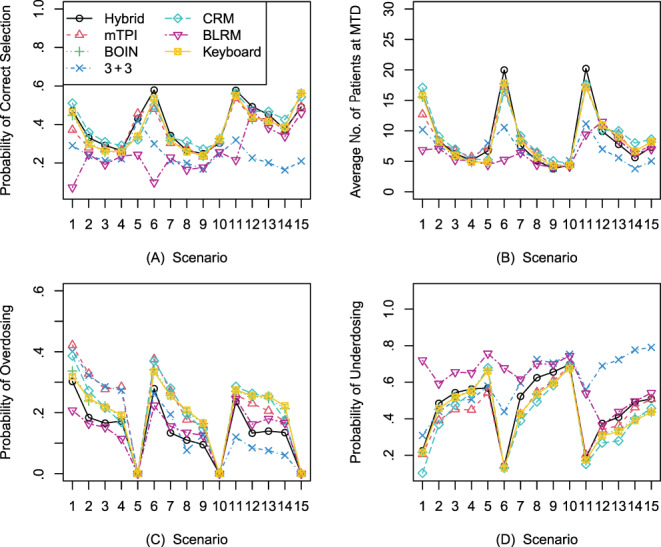
(A) Probability of correct MTD selection; (B) average number of participants treated at MTD; (C) probability of overdosing; (D) probability of underdosing. BLRM, Bayesian logistic regression model; BOIN, Bayesian optimal interval; CRM, continual reassessment method; MTD, maximum tolerated dose; mTPI, modified toxicity probability interval [Color figure can be viewed at wileyonlinelibrary.com]

Figure 1A,B shows the probability of correct selection and average number of participants treated at the MTD for the seven designs, respectively. In general, the 3 + 3 design yielded a lower correct selection rate compared to other model‐based and model‐assisted designs; thus, it treated fewer participants at the MTD. The correct selection rates and the average number of participants treated at the MTD for the hybrid, mTPI, Keyboard and BOIN designs were comparable across the 15 scenarios at three different target DLTs. The hybrid design performed better than the mTPI design in all scenarios except case 5. This was expected because the hybrid design is based on the mTPI design, with a logistic regression model added for the improvement of accuracy. The hybrid design performed better than the BOIN design and Keyboard design in all scenarios except scenario 15. The CRM had a better performance compared to other methods when the target DLT was 0.20 and for some of the scenarios when the target DLT was 0.25 or 0.30. The correct selection rate for the BLRM was low, especially when the first dose was the target MTD. This may have been due to the conservative overdosing control rule $\;\mathrm{Pr}\left(p_{1}>\delta_{2}|\text{data}\right)>0.25.$ The dose escalation tended to stop early with the imposed overdose control rule.

Overdosing control is the most relevant concern for protecting the safety of trial participants, and therefore it is strictly regulated by health authorities and ethics committees. Figure 1C,D shows the probability of overdosing and underdosing, respectively. The hybrid design had a very robust performance and yielded a relatively lower probability of overdosing across all 15 scenarios. It had a lower overdosing toxicity compared to the mTPI, Keyboard and BOIN designs for all 15 scenarios. The overdosing rate became lower for the 3 + 3 design as the target DLT got higher, because the 3 + 3 design targets a fixed DLT range. The risk of overdosing using the mTPI design was high when the target DLT was 0.20 and 0.25, and the risk of overdosing for the CRM, Keyboard and BOIN designs was high when the target DLT was 0.25 and 0.30. The BLRM had a lower overdosing rate when the target DLT was 0.20. The BLRM was the most conservative method, meaning that it was more likely to treat participants at a suboptimal dose when the target DLT was 0.20. The hybrid design had a slightly higher underdosing risk compared to other model‐assisted methods. The CRM had the lowest risk of underdosing in most of the scenarios.

In terms of safety, the hybrid design had a lower chance of selecting a toxic dose, as the MTD uniformly compared to other model‐based and model‐assisted designs with a reasonable underdosing percentage. Though the CRM outperformed other methods with regard to correct selection rate at a low MTD rate and risk of underdosing, it may aggressively select a toxic dose as the MTD.

### 4.2 Simulation for intermediate dose as MTD

In a real trial, the true MTD is usually not in the preselected provisional dose list. One advantage of the hybrid design is that an intermediate dose can be calibrated and selected as the MTD when the predicted probability of toxicity of the next dose exceeds the target DLT or when the toxicity level at the current dose level is over the target DLT level. We conducted simulations to compare the performance of the hybrid design to other approaches. A typical model‐assisted design chooses a dose among the doses in the provisional dose list. If the true MTD is not at exactly on one of the provisional dose list but between two adjacent doses, then a typical model‐assisted design has zero chance selecting a true MTD. However, the hybrid design has the chance selecting the true MTD due to its ability using an intermediate dose. Since not all methods are able to calibrate for an intermediate dose, it was not feasible to use overdosing and underdosing for comparison. We proposed a new measure: mean dose level deviation from the target MTD, for which the optimal result is the lowest mean dose level deviation, and is defined as:

$$\text{mean}\;\left(|\text{dose level selected from each simulation}-\text{target}\;\mathrm{MTD}\right).$$

In this new measure, the dose levels were ranked as 1, 2, 3, 4 or 5, from the lowest dose to the highest dose. If the target MTD was between dose Levels 2 and 3, the target MTD rank was 2.5. If an intermediate dose level between dose Levels 1 and 2 was selected by the hybrid design, the rank for the intermediate dose level would be 1.5. If dose escalation was terminated early, meaning that the first dose level was too toxic, a rank of 0.5 was assigned.

**FIGURE 2:**
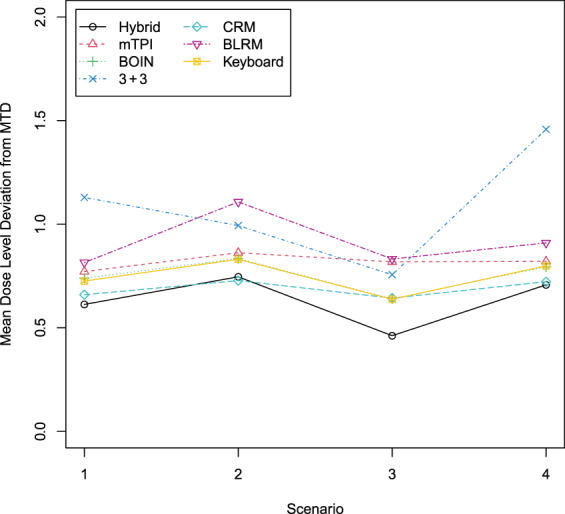
Mean dose level deviation from the true MTD. BLRM, Bayesian logistic regression model; BOIN, Bayesian optimal interval; CRM, continual reassessment method; MTD, maximum tolerated dose; mTPI, modified toxicity probability interval [Color figure can be viewed at wileyonlinelibrary.com]

Table 4B shows the four simulation scenarios with an intermediate dose as MTD. The target DLTs were 0.125, 0.20, 0.25 and 0.30, respectively. For each scenario, 10 000 trials were simulated. Note that only one intermediate dose between two adjacent doses was used in the simulation. The mean dose level deviation from the true MTD is displayed in Figure 2. The 3 + 3 design and the BLRM performed the worst among all the methods. The Keyboard and BOIN designs performed better than the mTPI design. The CRM and the hybrid design had a smaller deviation from the true MTD than other methods, but the hybrid design was better than the CRM in scenario 3.

In summary, with all simulated scenarios, the hybrid design had the most robust operating characteristics and the best overall performance among the selected designs. The hybrid design controlled overdosing effectively and gave an MTD estimate closest to the true MTD.

## 5 DISCUSSION

In this article, a hybrid design for dose‐finding oncology clinical trials was proposed. The design is a hybrid of the modified mTPI design and a dose‐toxicity model, and it is a hybrid of the Bayesian approach for each individual dose and the frequentist approach, combining the available information from all tested doses. This proposed hybrid design takes all the merits from the current existing designs and has a very robust performance. At each individual dose level, it diligently focuses on the DLT severity by using an existing Bayesian approach, such as the mTPI design. By combining the observed information from all the tested dose levels at the next step, it utilizes a dose‐toxicity model to further characterize and quantify the DLT level. The DLT information from both individual dose level and the model‐based quantification is used to recommend the dose‐escalation strategy. In principle, any model‐assisted design can be used in this hybrid design. Due to its more effectiveness in controlling the overdosing toxicity, a modified mTPI was used in the hybrid design. The hybrid design facilitates close communication and discussion for optimal dose decision‐making among the study team members, particularly the clinicians and the statisticians, which results in a deep understanding of the data leading to greater efficiency and more information for dose selection.

For a dose‐escalation trial, the most relevant question asked by health agencies and ethics committees is, “How well does the trial design control overdosing?” Our simulation results indicate that the proposed hybrid design has the best overdosing toxicity control rate among the commonly used scenarios. Thus, the recommended dose from the hybrid design has fewer safety concerns. In practice, it is commonly challenging to have the MTD as exactly one of the prelisted provisional dose levels. The hybrid design is able to calibrate the MTD and recommend an intermediate dose level, if needed, using the dose‐toxicity model. The simulation results indicate that the recommended MTD from the hybrid design is much closer to the true MTD among the commonly used scenarios. Thus, this hybrid design has a high probability of more precisely selecting an optimal dose for Phase II and Phase III trials to reduce attrition rates in late‐phase oncology clinical development.

In practice, a provisional dose level list is usually carefully selected before the trial begins. If, after the highest dose level in the provisional list is tested, there is no DLT observed and no clear efficacy‐related signal, then the dose‐escalation procedure may be continued, with  additional participants enrolled at a higher dose level. At the end of the dose‐escalation procedure, the DLT rates at all tested dose levels are estimated based on the dose‐toxicity model. The dose with an estimated DLT rate closest to targeted toxicity rate will be treated as the MTD. However, the totality of the available data, such as the emerging safety, PK, PD and other biomarker information, is considered before deciding on the recommended dose for cohort expansion and Phase II trial. To help implement the proposed hybrid design, an R‐shiny tool (https://fzh223.shinyapps.io/HybridModel/) has been developed and is freely available to guide clinicians in every step of dose‐finding process.

## 6 LIMITATIONS

The hybrid design is built on top of a modified mTPI, and it requires pooling all available information accumulated and running a dose‐toxicity model for determining the next dose level. However, a web‐based, freely available R‐shiny app for the hybrid design was developed for the simplicity of use.

## 7 FUTURE DIRECTIONS

The current hybrid design only deals with the dose escalation for monotherapy. In recent years, more and more studies seem to focus on the combination of two novel agents. As such, we are working to expand the method for the dose escalation of two novel drugs in combination.

## 8 CONCLUSIONS

The hybrid design is a hybrid of the modified mTPI design and a dose‐toxicity model, and it is a hybrid of the Bayesian approach for each individual dose and the frequentist approach for combining the available information from all tested doses. The hybrid design takes all the merits from the current existing designs and has a very robust performance. The hybrid design controls the overdosing toxicity well and leads to a recommended dose closer to the true MTD. With the integration of all available data and interpolation of information across dose groups on top of the modified mTPI, the hybrid design leads to more accuracy and efficiency for dose selection. The design procedure facilitates close communication for more robust dose decision‐making among the study team members, particularly between the clinician and the statistician, which results in a deep understanding of the data leading to greater efficiency and more information for dose selection.

## AUTHOR CONTRIBUTIONS

The work reported in the paper has been performed by the authors, unless clearly specified in the text. Jason J. Z. Liao contributed to the conceptualization, methodology, formal analysis, software and writing. Feng Zhou and Heng Zhou contributed to the methodology, software and writing. Lilli Petruzzelli, Kevin Hou and Ekaterine Asatiani contributed to the methodology and writing.

## CONFLICT OF INTEREST

The authors declare no potential conflicts of interest.

## Supporting information


**Appendix S1** Supporting Information

## Data Availability

The datasets generated and/or analyzed during the current study are available upon reasonable request from the corresponding author (email: jliao@incyte.com).
