# Supplementary material for: A hybrid design for dose‐finding oncology clinical trials
Source: Int J Cancer. 2022 Jul 21;151(9):1602–10. doi: 10.1002/ijc.34203 (PMC10084431; doi:10.1002/ijc.34203)
Supplement: Supplementary file 1 — Appendix S1 Supporting Information [file IJC-151-1602-s001.pdf]

Data Supplement

A hybrid design for dose-finding oncology clinical trials

Jason J.Z. Liao | Feng Zhou | Heng Zhou | Lilli Petruzzelli | Kevin Hou | Ekaterine Asatiani

1. SUPPLEMENTARY 1: The analysis results from the trial example using the developed R-shiny tool (https://fzh223.shinyapps.io/HybridModel/)
2. SUPPLEMENTARY 2: R-code for hybrid design

SUPPLEMENTARY 1 The analysis results from the trial example using the developed R-shiny tool (*https://fzh223.shinyapps.io/HybridModel/*), where the left panel lists the data entries and parameter settings, and the right panel shows the dose-escalation decision.

Hybrid Design - Dynamic user interface

Enter a vector of dose levels (comma delimited)

0.4,1.2,3.5,10,25,75

Enter a vector of number of DLTs on each dose level (comma delimited)

0,0,0,0,1,2

Enter a vector of number of patients assigned to each dose level (comma delimited)

1,1,1,3,5,6

Current Dose

75

Enter a vector of Next Dose (comma delimited)

150,70

Target dose limiting toxicity (phi):

0 — 0.33 — 1

0 0.1 0.2 0.3 0.4 0.5 0.6 0.7 0.8 0.9 1

Summary

```
$mTPIboundary

Number of patients treated  1 2 3 4 5 6 7 8 9 10 11 12 13 14 15
Escalate if # of DLT <=     0 0 0 0 0 1 1 1 1  2  2  2  2  2  3
Deescalate if # of DLT >=   1 2 2 3 3 4 4 5 5  6  6  6  7  7  8
Eliminate if # of DLT >=   NA 2 3 3 4 4 5 5 6  6  7  7  8  8  8

$decision_table
   1    2    3    4    5    6    7    8    9    10   11   12   13   14   15
0  "E"  "E"  "E"  "E"  "E"  "E"  "E"  "E"  "E"  "E"  "E"  "E"  "E"  "E"  "E"
1  "D"  "S"  "S"  "S"  "S"  "E"  "E"  "E"  "E"  "E"  "E"  "E"  "E"  "E"  "E"
2  NA   "DU" "D"  "S"  "S"  "S"  "S"  "S"  "S"  "E"  "E"  "E"  "E"  "E"  "E"
3  NA   NA   "DU" "DU" "D"  "S"  "S"  "S"  "S"  "S"  "S"  "S"  "S"  "S"  "E"
4  NA   NA   NA   "DU" "DU" "DU" "D"  "S"  "S"  "S"  "S"  "S"  "S"  "S"  "S"
5  NA   NA   NA   NA   "DU" "DU" "DU" "DU" "D"  "S"  "S"  "S"  "S"  "S"  "S"
6  NA   NA   NA   NA   NA   "DU" "DU" "DU" "DU" "DU" "D"  "D"  "S"  "S"  "S"
7  NA   NA   NA   NA   NA   NA   "DU" "DU" "DU" "DU" "DU" "DU" "D"  "D"  "S"
8  NA   NA   NA   NA   NA   NA   NA   "DU" "DU" "DU" "DU" "DU" "DU" "DU" "DU"
9  NA   NA   NA   NA   NA   NA   NA   NA   "DU" "DU" "DU" "DU" "DU" "DU" "DU"
10 NA   NA   NA   NA   NA   NA   NA   NA   NA   "DU" "DU" "DU" "DU" "DU" "DU"
11 NA   NA   NA   NA   NA   NA   NA   NA   NA   NA   "DU" "DU" "DU" "DU" "DU"
12 NA   NA   NA   NA   NA   NA   NA   NA   NA   NA   NA   "DU" "DU" "DU" "DU"
13 NA   NA   NA   NA   NA   NA   NA   NA   NA   NA   NA   NA   "DU" "DU" "DU"
14 NA   NA   NA   NA   NA   NA   NA   NA   NA   NA   NA   NA   NA   "DU" "DU"
15 NA   NA   NA   NA   NA   NA   NA   NA   NA   NA   NA   NA   NA   NA   "DU"
```

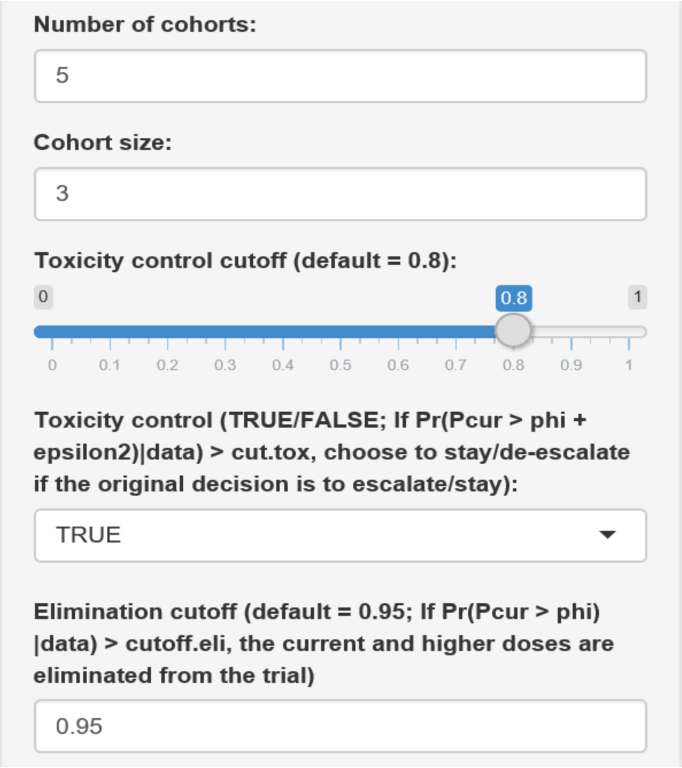


```
$decision
[1] "Deescalate"

$dose_toxicity_and_action_table
                        Dose    Estimated Toxicity Action
Current Dose            "75"    "0.355"            "Not Selected"
Prior Intermediate Dose "71.42" "0.33"             "Selected"
Prior Dose              "25"    "0.102"            "Not Selected"
Next Intermediate Dose  "NA"    "NA"               "Not Selected"
Next Dose               "150"   "0.854"            "Not Selected"
Next Dose               "70"    "0.32"             NA
```

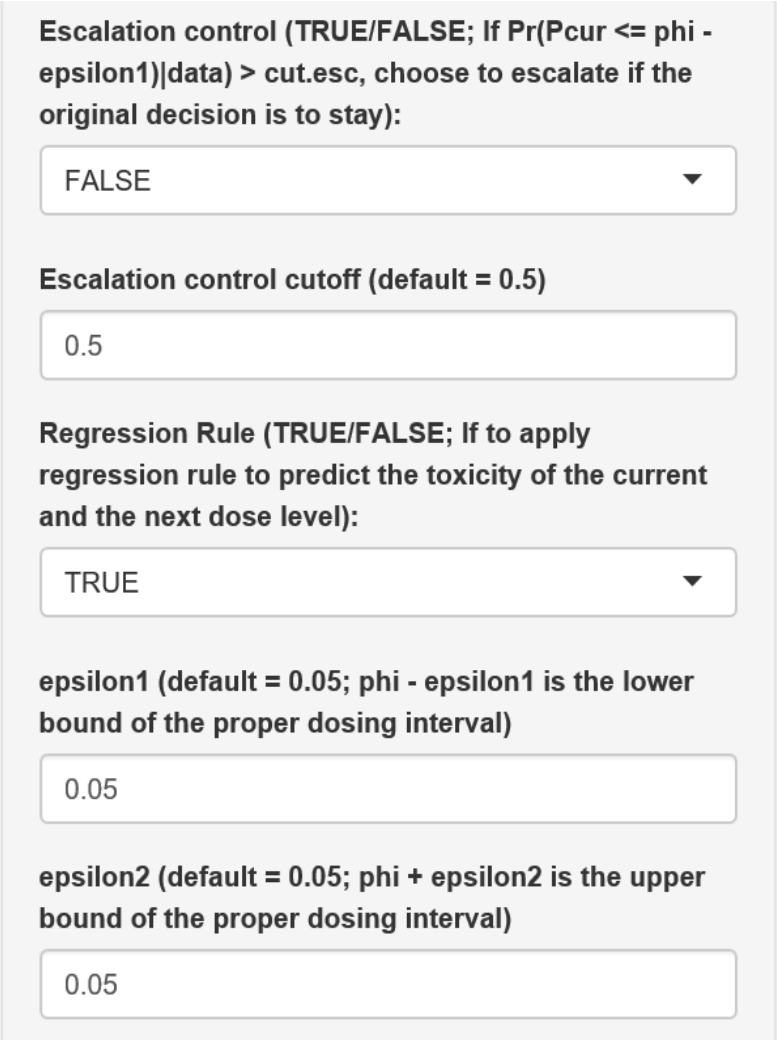
Escalation control (TRUE/FALSE; If Pr(Pcur <= phi - epsilon1)|data) > cut.esc, choose to escalate if the original decision is to stay):
FALSE
Escalation control cutoff (default = 0.5)
0.5
Regression Rule (TRUE/FALSE; If to apply regression rule to predict the toxicity of the current and the next dose level):
TRUE
epsilon1 (default = 0.05; phi - epsilon1 is the lower bound of the proper dosing interval)
0.05
epsilon2 (default = 0.05; phi + epsilon2 is the upper bound of the proper dosing interval)
0.05

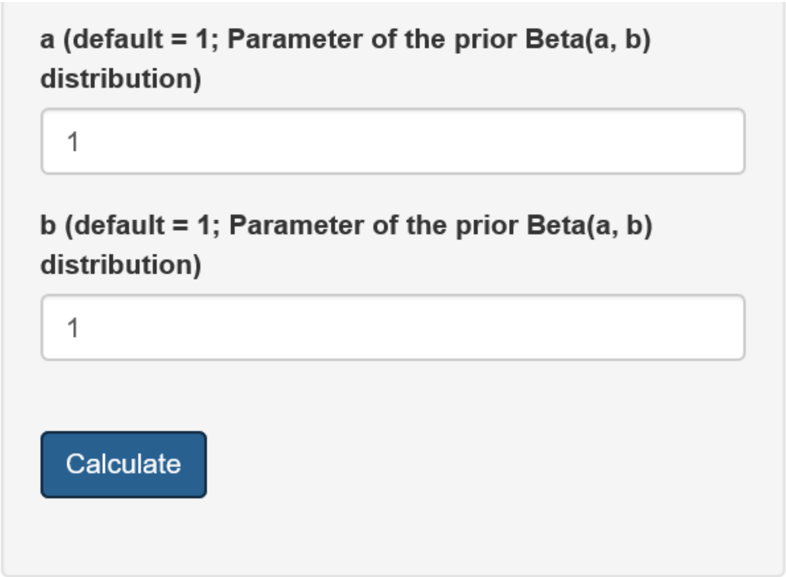

SUPPLEMENTARY 2 R-code for hybrid design

```
get.boundary.mtpi <- function(target, ncohort, cohortsize, eps1=0.05, eps2=0.05, a=1, b=1, cutoff.eli=0.95,
                              tox.control=FALSE, cut.tox=0.8, esc.control=FALSE, cut.esc=0.5) {

 ### simple error checking
 if(target<0.05) {cat("Error: the target is too low! \n"); return();}
 if(target>0.6)  {cat("Error: the target is too high! \n"); return();}

 sampsize = ncohort * cohortsize
 decision_table = matrix(NA,nrow=sampsize+1,ncol=sampsize)
 for (ntr in 1:sampsize)
 {
  elim=0
  for (ntox in 0:ntr)
```

```
    {
      q1 <- (1-pbeta(eps2+target, ntox+a, ntr-ntox+b))/(1-eps2-target)
      q2 <- (pbeta(eps2+target, ntox+a, ntr-ntox+b) - pbeta(target-eps1, ntox+a, ntr-ntox+b))/(eps2+eps1)
      q3 <- (pbeta(target-eps1, ntox+a, ntr-ntox+b)/(target-eps1))
      if (q3==max(q1,q2,q3)) decision_table[ntox+1,ntr] <- ifelse(tox.control,ifelse(1-pbeta(eps2+target, ntox+a, ntr-
ntox+b)>cut.tox,"S","E"),"E")
      if (q2==max(q1,q2,q3)) {
        temp1 <- ifelse(tox.control,ifelse(1-pbeta(eps2+target, ntox+a, ntr-ntox+b)>cut.tox,"D","S"),"S")
        temp2 <- ifelse(esc.control,ifelse(pbeta(target-eps1, ntox+a, ntr-ntox+b)>cut.esc,"E","S"),"S")
        if(temp1=="D") decision_table[ntox+1,ntr] <- "D"
        else if(temp2=="E") decision_table[ntox+1,ntr] <- "E"
        else decision_table[ntox+1,ntr] <- "S"
      }
      if (q1==max(q1,q2,q3)) decision_table[ntox+1,ntr] <- "D"
      if (1-pbeta(target, ntox+a, ntr-ntox+b)>cutoff.eli) {elim=1;break;}
    }
    if (elim==1)
    {
      if (decision_table[ntox+1,ntr]=="D") {decision_table[(ntox+1):(ntr+1),ntr] <- rep("DU",ntr-ntox+1)}
      else {decision_table[(ntox+2):(ntr+1),ntr] <- rep("DU",ntr-ntox)}
    }
  }
  colnames(decision_table) <- 1:sampsize
  rownames(decision_table) <- 0:sampsize

  boundary = matrix(NA, nrow=4, ncol=sampsize)
  boundary[1,] = 1:sampsize
  for(i in 1:sampsize)
  {
    boundary[2,i] = max(which(decision_table[,i]=="E"))-1
    if (length(which(decision_table[,i]=="D"))) {boundary[3,i] = min(which(decision_table[,i]=="D"))-1}
    else if (length(which(decision_table[,i]=="DU"))) {boundary[3,i] = min(which(decision_table[,i]=="DU"))-1}
    if (length(which(decision_table[,i]=="DU"))) {boundary[4,i] = min(which(decision_table[,i]=="DU"))-1}
```

```
  }
  colnames(boundary) <- c(rep("", sampsize))
  rownames(boundary) <- c("Number of patients treated", "Escalate if # of DLT <=", "Deescalate if # of DLT >=", "Eliminate
if # of DLT >=")

  out=list()
  if(cohortsize>1){
    out=list(
      boundary_tab=boundary[, (1:ncohort)*cohortsize],
      full_boundary_tab=boundary,decision_table=decision_table)
  }else
    out=list(full_boundary_tab=boundary[, (1:ncohort)*cohortsize],decision_table=decision_table)

  class(out) <- "mtpi"
  return(out);
}


Hybrid <- function(ndose, nDLT, npts, currdose, nextdose=0, target, ncohort, cohortsize, eps1=0.05, eps2=0.05, a=1, b=1,
cutoff.eli=0.95,
                   tox.control=TRUE, cut.tox=0.8, esc.control=FALSE, cut.esc=0.5, regrule=1){

  mTPIout <- get.boundary.mtpi(target, ncohort, cohortsize, eps1, eps2, a, b, cutoff.eli, tox.control, cut.tox, esc.control, cut.esc)

  mTPIbound <- mTPIout$full_boundary_tab
  mTPIbound[4,-which(mTPIbound[4,]<10000)] <- 9999
  decision_table <-  mTPIout$decision_table

  currDLT =nDLT[which(ndose==currdose)]

  #decision from the mTPI: decision1

  if (currDLT >= mTPIbound[4,npts[which(ndose==currdose)]]) {decision1 <- "Eliminate"}
```

```
  else if (currDLT >= mTPIbound[3,npts[which(ndose==currdose)]] & currDLT < 
mTPIbound[4,npts[which(ndose==currdose)]]) {decision1 <- "Deescalate"}
  else if (currDLT > mTPIbound[2,npts[which(ndose==currdose)]] & currDLT < 
mTPIbound[3,npts[which(ndose==currdose)]]){decision1 <- "Stay"}
  else if (currDLT <= mTPIbound[2,npts[which(ndose==currdose)]]){decision1 <- "Escalate"}

  mTPIbound[4, -which(mTPIbound[4,]<9999)] <- NA

  ## Logstic regression model

  nlength <- length(ndose)
  ptox <- NULL
  dosevec <- NULL

  for (i in 1:nlength){
    ptox <- append(ptox, c(rep(0, (npts[i]-nDLT[i])), rep(1, nDLT[i])))
    dosevec <- append(dosevec, rep(ndose[i], npts[i]))
  }

  warnmsg <- 0
  BLRMrule <- 1
  pval <- 0

  model <- glm(ptox ~ dosevec, family = "binomial")
  warnmsg <- has_warning(model <- glm(ptox ~ dosevec, family = "binomial"))

  if (is.na(model$coeff[2])==FALSE &  is.na(model$coeff[1])==FALSE){
    if (abs(model$coeff[2])> 0.0001 & abs(model$coeff[1])> 0.0001){
      pval <- hoslem.test(ptox, fitted(model))$p.value
    }
  }

  # Use mTPI when the logistic model does not fit well
```

```
if (is.na(model$coeff[2])==TRUE | warnmsg==1 | is.na(pval)==TRUE){BLRMrule <- 0}
else if (is.na(model$coeff[2])==TRUE | warnmsg==1 | pval < 0.05){BLRMrule <- 0}
else if (is.na(model$coeff[2])==FALSE & model$coeff[2]<0){BLRMrule <- 0}

if (regrule==0){BLRMrule <- 0}

if (BLRMrule==1){

  ## Estimate toxicity for current, next, next intermediate, prior, prior intermediate dose

  action <- c(rep("Not Selected", 5),rep(NA,length(nextdose)-1))

  currptox <- predict(model, data.frame(dosevec = currdose), type = "response")
  currvec <- round(cbind(currdose, currptox),3)

  nextptox <- predict(model, data.frame(dosevec = nextdose), type = "response")
  nextvec <- round(cbind(nextdose, nextptox),3)

  nextdose0 = -(model$coeff[1]+log((1-target)/target))/model$coeff[2]
  nextptox0 = target

  intervec <- round(cbind(nextdose0, nextptox0),3)

  priordose = ndose[max(which(ndose==currdose)-1,1)]
  priorptox = predict(model, data.frame(dosevec = priordose), type = "response")
  priorvec = round(cbind(priordose, priorptox),3)

  priordose0 = -(model$coeff[1]+log((1-(target))/(target)))/model$coeff[2]
  priorptox0 = target
  priorvec0 = round(cbind(priordose0, priorptox0),3)

  nextptox11 <- predict(model, data.frame(dosevec = 2*currdose), type = "response")
```

```
if ((min(nextptox[1],nextptox11) > 0.95) & priorptox < 0.05 ){BLRMrule <- 0}

## mTPI suggests escalate
if (decision1 == "Escalate"){
  if (currptox <= target & nextptox[1] <= target){
    decision2 <- decision1
    action[5] <- "Selected"
    priorvec0 <- cbind("NA","NA")
    intervec <- cbind("NA","NA")
  }
  else if (currptox <= target & nextptox[1] > target){
    if (nextptox0 > target){
      decision2 <- "Stay"
      action[1] <- "Selected"
      priorvec0 <- cbind("NA","NA")
    }
    else {
      decision2 <- decision1
      action[4] <- "Selected"
      priorvec0 <- cbind("NA","NA")
    }
  }
  else if (currptox > target){
    decision2 <- "Stay"
    action[1] <- "Selected"
    priorvec0 <- cbind("NA","NA")
    intervec <- cbind("NA","NA")
  }
}

## mTPI suggests stay
else if (decision1 == "Stay"){
  if (currptox <= target){
```

```
    decision2 <- decision1
    action[1] <- "Selected"
    priorvec0 <- cbind("NA","NA")
    intervec <- cbind("NA","NA")
  }
  else if (currptox > target){
    if (priorptox0 <= target){
      action[2] <- "Selected"
      intervec <- cbind("NA","NA")
    }
    else {
      action[3] <- "Selected"
      intervec <- cbind("NA","NA")
    }
    decision2 <- "Deescalate"

  }
}

## mPTI suggests deescalate
else if (decision1  == "Deescalate" || decision1 == "Eliminate"){
  if (priorptox0 <= target){
    action[2] <- "Selected"
    intervec <- cbind("NA","NA")
  }
  else {
    action[3] <- "Selected"
    intervec <- cbind("NA","NA")
  }
  decision2 <- decision1
}

## Output table
```

```
    dosetab <- rbind(currvec, priorvec0, priorvec, intervec, nextvec)
    nextdoselb <- rep("Next Dose",nrow(nextvec))
    doselevel <- c("Current Dose","Prior Intermediate Dose", "Prior Dose", "Next Intermediate Dose", nextdoselb)
    dosetab <- cbind(doselevel, dosetab, action)
    colnames(dosetab) <- c("Dose Level","Dose", "Estimated Toxicity","Action")
    rownames(dosetab) <- seq(1:nrow(dosetab))
  }

  if (BLRMrule == 0){
    decision2 <- decision1
  }

  out=list()
  if (BLRMrule==0){
    out=list(
      mTPIboundary = mTPIbound,
      decision_table = decision_table,
      decision = decision2
    )
  }

  else if (BLRMrule==1){
    out=list(
      mTPIboundary = mTPIbound,
      decision_table = decision_table,
      decision = decision2,
      dose_toxicity_and_action_table = dosetab
    )
  }
  return(out);
}
```
